# Antimicrobial Resistance and Molecular Characterization of *Citrobacter* spp. Causing Extraintestinal Infections

**DOI:** 10.3389/fcimb.2021.737636

**Published:** 2021-08-27

**Authors:** Liyun Liu, Ling Zhang, Haijian Zhou, Min Yuan, Dalong Hu, Yonglu Wang, Hui Sun, Jianguo Xu, Ruiting Lan

**Affiliations:** ^1^State Key Laboratory of Infectious Disease Prevention and Control, National Institute for Communicable Disease Control and Prevention, Chinese Center for Disease Control and Prevention, Research Units of Discovery of Unknown Bacteria and Function (2018RU010), Chinese Academy of Medical Sciences, Beijing, China; ^2^Microbiology Department, Maanshan Center for Clinical Laboratory, Ma’anshan, China; ^3^Microbiology Department, Maanshan Center for Disease Control and Prevention, Ma’anshan, China; ^4^School of Biotechnology and Biomolecular Sciences, University of New South Wales, Sydney, NSW, Australia

**Keywords:** *Citrobacter* spp., sequence types, multidrug resistance, *gyrA*, cytotoxicity

## Abstract

**Objectives:**

This prospective study was carried out to investigate molecular characteristics and antimicrobial susceptibility patterns of *Citrobacter* spp. from extraintestinal infections.

**Methods:**

Forty-six clinical *Citrobacter* spp. isolates were isolated from hospital patients with extraintestinal infections and analyzed by multilocus sequence typing (MLST) using seven housekeeping genes. Antimicrobial susceptibility testing was performed by disk diffusion method according to the Clinical and Laboratory Standards Institute (CLSI) recommendations. Adhesion and cytotoxicity to HEp-2 cells were assessed.

**Results:**

The 46 clinical *Citrobacter* spp. isolates were typed into 38 sequence types (STs), 9 of which belonged to four clonal complexes (CCs). None of the isolates shared the same ST or CCs with isolates from other countries or from other parts of China. Over half of the isolates were multidrug-resistant (MDR), with 17/26 C*. freundii*, 5/6 C*. braakii*, and 3/14 C*. koseri* isolates being MDR. Moreover, four isolates were carbapenem resistant with resistance to imipenem or meropenem. Among eight quinolone resistant *C. freundii*, all had a mutation in codon 59 (Thr59Ile) in quinolone resistance determining region of the *gyrA* gene. Only a small proportion of the isolates were found to be highly cytotoxic and adhesive with no correlation to sample sources.

**Conclusions:**

There was a diverse range of *Citrobacter* isolates causing extraintestinal infections and a high prevalence of MDR.

## Introduction

*Citrobacter* spp. are facultative anaerobic Gram negative bacteria within the family Enterobacteriaceae. *Citrobacter* spp. have been associated with nosocomial infections involving the urinary tract, liver, biliary tract, peritoneum, intestines, bone, respiratory tract, endocardium, wounds, soft tissue, meninges, and the bloodstream (Khorasani et al., 2008; Kumar et al., 2013; Liu et al., 2018b). C. *freundii* is the most frequently isolated *Citrobacter* species from a range of infections (Khorasani et al., 2008) and has also caused small outbreaks in healthcare settings (Nada et al., 2004; Mohanty et al., 2007; Samonis et al., 2009; Bai et al., 2012). *C. koseri* can cause meningitis and brain abscesses in neonates and central nervous system (CNS) infections in head trauma, facial fractures, post neurosurgical procedures, or immunocompromised individuals (Vaz Marecos et al., 2012; Chao et al., 2013; Lechowicz et al., 2017; Reyes et al., 2017). *C. braakii* has been reported to cause bacteriemia (Lai et al., 2010; Hirai et al., 2016; Oyeka and Antony, 2017).

Emergence of multidrug-resistant (MDR) *Citrobacter* strains is an increasing concern (Khorasani et al., 2008). MDR *C. freundii* strains have been associated with a higher rate of in-hospital mortality compared to susceptible strains (Deal et al., 2007). MDR *Citrobacter* spp. with production of β-lactamase (Amp-C), broad-spectrum β-lactamase, extended-spectrum β-lactamase (ESBL), or even carbapenemase has been reported by several international surveillance programs (Wang et al., 2000; Mohanty et al., 2007; Zhang et al., 2008; Samonis et al., 2009; Kanamori et al., 2011; Lee et al., 2015; Liu et al., 2018b). It has been reported that 39–48% of *C. freundii* isolates were resistant to broad-spectrum cephalosporins (ceftriaxone, ceftazidime), piperacillin, and piperacillin/tazobactam (Khorasani et al., 2008). Moreover, a few studies have reported *C. freundii* harboring carbapenemases, particularly metallo-β-lactamases (MBLs) or *Klebsiella pneumoniae* carbapenemase (KPC) types (Weile et al., 2007; Protonotariou et al., 2008; Zhang et al., 2008; Gaibani et al., 2013; Schweizer et al., 2019; Gobeille Paré et al., 2020; Räisänen et al., 2021). Quinolone resistance determinant including *qnr* and *aac(6’)-Ib-cr* genes have been reported in *Citrobacter* spp. (Park et al., 2007; Zhang et al., 2012). Numerous *qnrB* alleles have been detected, and about 40 *qnrB* variants are located on the chromosome of *Citrobacter* spp., especially *C. freundii* (Jacoby et al., 2014, Liao et al., 2015). Fluoroquinolone resistance is associated with mutations in *gyrA* and *parC* genes (Minarini and Darini, 2012). Mutations in *gyrA* were found in fluoroquinolone resistant *C. freundii* isolates (Weigel et al., 1998; Minarini and Darini, 2012).

Studies on *Citrobacter* spp. from extraintestinal infections have been mostly focused on antibiotic resistance, and little is known about their genetic diversity and virulence properties. *Citrobacter* spp. can be isolated from fecal samples of healthy individuals and can also cause food-borne infections (Tassew et al., 2010; Bai et al., 2012; Ifeadike et al., 2012; Minarini and Darini, 2012; Liu et al., 2017; Liu et al., 2018a; Liu et al., 2020). The possible source of strains causing extraintestinal infections and their relationships to strains from other infections and other sources have not been well studied. In our previous studies, we analyzed *Citrobacter* isolates from diarrheal patients, foods, and environment in China (Bai et al., 2012; Liu et al., 2017; Liu et al., 2018a; Liu et al., 2020). We found high diversity of *Citrobacter* strains from these sources in sequence types (STs), antibiotic resistance profiles, and virulence properties (Liu et al., 2017; Liu et al., 2018a; Liu et al., 2020). In this study, we collected *Citrobacter* spp. isolates from extraintestinal infections of inpatients in Maanshan people's hospital, Anhui Province, China and examined these isolates by multilocus sequence typing (MLST), antibiotic resistance profiling, and *in vitro* virulence testing to obtain an insight into their genetic diversity, antibiotic resistance, and virulence.

## Materials and Methods

### *Citrobacter* Isolates

Forty-six *Citrobacter* spp. isolates were obtained from 26 urine, 15 sputum, 2 bile, 2 secretion, and 1 blood samples from 2014 to 2018 in Maanshan people hospital, Anhui Province, China. The 26 urine samples included 16 C*. freundii*, 1 *C. braakii*, and 9 C*. koseri* isolates; the 15 sputum samples contained 8 C*. freundii*, 3 *C. braakii*, and 4 C*. koseri* isolates; 2 bile samples contained *C. freundii* isolates; 2 secretion samples contained *C. braakii* isolates; 1 blood sample had *C. koseri* isolates. No other pathogens were isolated from these clinical specimens with the exception of a sputum sample where *Citrobacter* is the predominant pathogen. The identity of each isolate was determined using API 20E test strips (bioMérieux, La Balme les Grottes, France) at the time of isolation, and they were stored as glycerol stocks at -80°C. Bacteria were grown in Luria-Bertani (LB) broth or on LB and Mueller–Hinton agar plates (pH 7.4) at 37°C.

### Antimicrobial Susceptibility Testing

Antimicrobial susceptibility testing was carried out using the disk diffusion method according to CLSI recommendations (Clinical and Laboratory Standards Institute, 2016). We tested the following 20 antimicrobial agents: ampicillin (AMP, 10 μg), cefotaxime (CTX, 30 μg), ceftazidime (CAZ, 30 μg), cefepime (FEP, 30 μg), cefoxitin (FOX, 30 μg), imipenem (IMP, 10 μg), aztreonam (ATM, 30 μg), meropenem (MEM, 10 μg), nalidixic acid (NA, 30 μg), ciprofloxacin (CIP, 5 μg), levofloxacin (LEV, 5 μg), gentamicin (CN, 10 μg), amikacin (AK, 30 μg), streptomycin (S, 10 μg), kanamycin (K, 30 μg), tetracycline (TE, 30 μg), doxycycline (DO, 30 μg), chloramphenicol (C, 30 μg), trimethoprim/sulfamethoxazole (SXT, 25 μg), and azithromycin (AZM, 15 μg) (Oxoid, Hampshire, UK). Quality control was performed using the reference strain *E. coli* ATCC 25922. Results were used to classify isolates as being resistant or susceptible to a particular antibiotic comparing with the standard reference values (Clinical and Laboratory Standards Institute, 2016).

For fluoroquinolones resistant isolates, susceptibility testing to quinolones including nalidixic acid (NA), ciprofloxacin (CIP), norfloxacin (NOR), and levofloxacin (LEV) was carried out using the broth microdilution method according to CLSI recommendations, as previously described (Liu et al., 2017). Minimum inhibitory concentration (MIC) results were interpreted according to the European Committee on Antimicrobial Susceptibility Testing (EUCAST) guidelines. Quality control for MICs was performed using the reference *E. coli* ATCC 25922.

### PCR Amplification and Sequencing

All the isolates were screened for *qnrA*, *qnrB*, *qnrS*, *qnrC*, *qnrD*, *aac(6’)-Ib-cr*, and *qepA* genes by PCR using previously published primers and protocols (Liu et al., 2020). All primers were synthesized by Shanghai Sangon Biological Engineering Technology and Services (Shanghai, China). Positive PCR products were confirmed by sequencing.

### MultiLocus Sequence Typing

The seven housekeeping genes, including *aspC*, *clpX*, *fadD*, *mdh*, *arcA*, *dnaG*, and *lysP*, were typed by PCR using previously published primers and protocols (Liu et al., 2020). Alleles and STs were assigned using the MLST database (http://pubmlst.org/cfreundii/).

PHYLOViZ version 2.0 (Francisco et al., 2012; Luo et al., 2021), using the goeBURST algorithm, was used to calculate and visualize clonal complexes (CCs) between the STs of the isolates. MEGA X (Kumar et al., 2018) was used to construct phylogenetic trees using the neighbor-joining algorithm with the default parameters based on the concatenated sequences of the seven housekeeping genes. Bootstraps with 1000 replicates was performed to evaluate the robustness of the branches of the tree.

### *In Vitro* Adhesion and Cytotoxicity Assays

*In vitro* adhesion to host cells was performed as previously described (Liu et al., 2017). An adhesion index (<1; >1 and <50; >50) describing the mean number of bacteria per HEp-2 after examination of 10 visual fields was determined (Liu et al., 2017). Infections were repeated three times in duplicate.

The lactate dehydrogenase (LDH) released by the HEp-2 cells was determined using the Cytotox 96 kit (Promega) according to the manufacturer’s instructions. The relative amount of cytotoxicity was expressed as previously described (Liu et al., 2017). All experiments were performed three times in duplicate.

### Statistical Analysis

SPSS software version 13.0 (SPSS Inc., Chicago, IL, USA) was used to conduct all statistical comparisons. A nonparametric test (Mann–Whitney U-test) was employed to compare the different groups. Two-tailed p-value of 0.05 or less was considered to be statistically significant.

## Results

### Clinical Characteristics of the Patients

**Figure 1:**
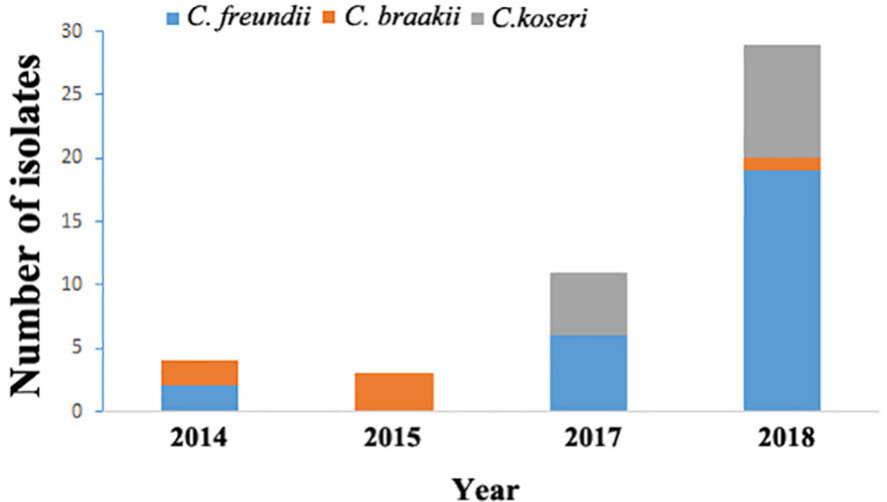
Distribution of *Citrobacter* isolates by year in Maanshan people’s hospital, Anhui Province, China from 2014 to 2018.

From September 2014 through August 2018, 46 cases of *Citrobacter* infections were identified and the distribution of isolates by year was presented in **Figure 1**. Among them, 26 (56.5%) cases had infections caused by *C. freundii* and 20 had non-*C. freundii* infections (6 of *C. braakii* and 14 of *C. koseri*). There was no clustering of cases or suspected outbreak during the study period. The median age was 65.1 years with a range of 0.1–91. Urinary system disease (16/46, 34.8%) was the common underlying disease among the patients (**Table 1**).

**Table 1:** Clinical characteristics of the patients with *Citrobacter* infections.

	Number and percentage of patients or isolates of a given species			
	Total (N = 46)	*C. freundii* (N = 26)	*C. braakii* (N = 6)	*C. koseri* (N = 14)
Age, year, median (IQR)	65.1 (0.1–91.0)	69.1 (37–89.0)	56.9 (0.1–78)	61.2 (0.1–91)
Sex, male	33 (71.7)	19 (73.1)	5 (83.3)	9 (64.3)
Urinary system disease	16 (34.8)	13 (25.0)	1 (16.7)	2 (14.3)
Respiratory system disease	4 (8.7)	1 (3.8)	1 (16.7)	2 (14.3)
Cardiovascular disease	3 (6.5)	0	0	3 (21.4)
Hepatobiliary tract disease	5 (10.9)	4(15.4)	0	1 (7.1)
Brain diseases	3 (6.5)	1 (3.8)	2 (33.3)	0
Diabetes mellitus	2 (4.3)	1 (3.8)	0	1 (7.1)
Pelvis fracture	1 (2.2)	1 (3.8)	0	0
Myelitis	1 (2.2)	0	0	1 (7.1)
Non-Hodgkin lymphoma	1 (2.2)	1 (3.8)	0	0
Unknown	10 (21.7)	4 (15.4)	2 (33.3)	4 (28.6)

### Multilocus Sequence Typing of *Citrobacter Isolates*

**Figure 2:**
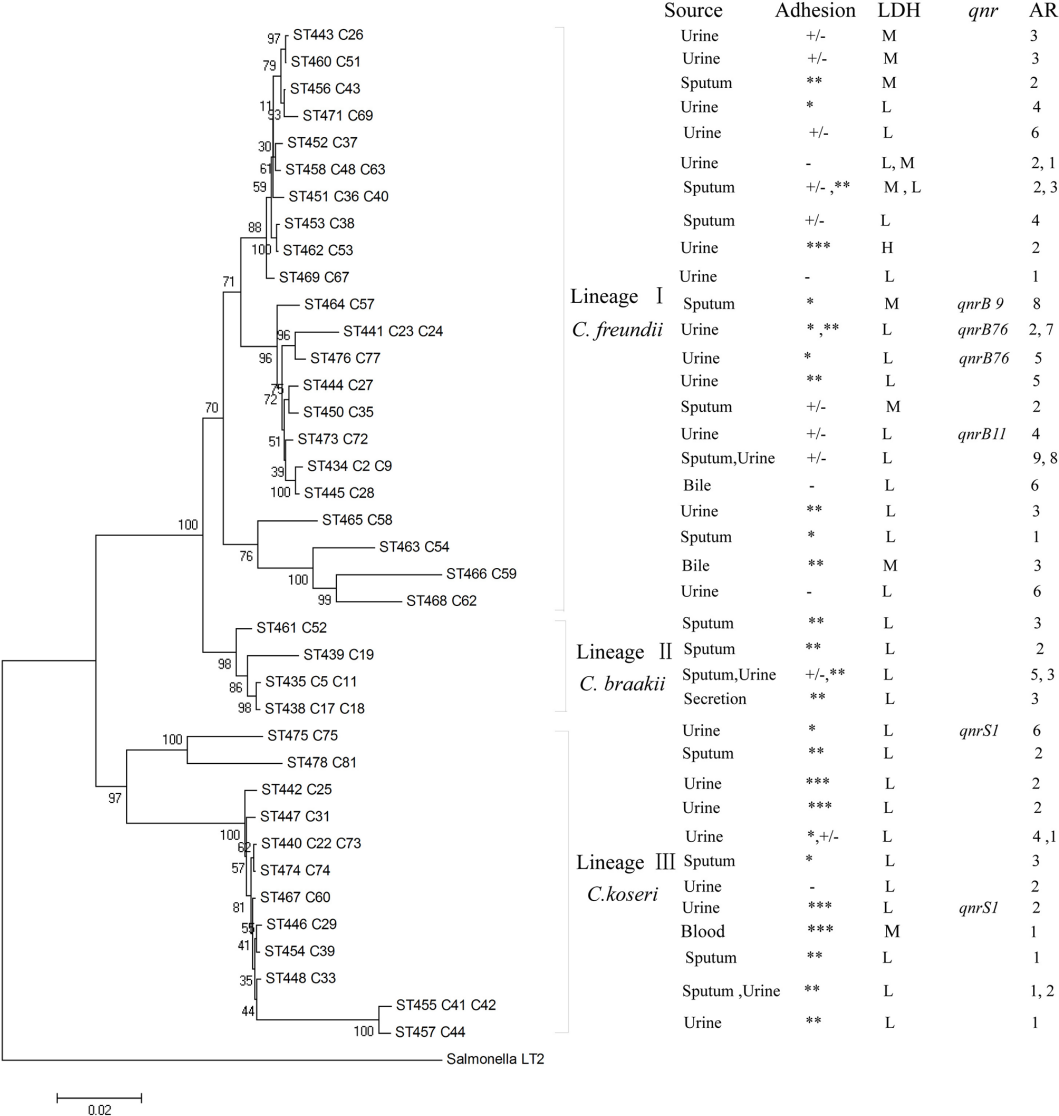
Phylogenetic relationships of the 46 *Citrobacter* isolates as determined by MLST data. The isolates were first marked with ST number followed by isolate name or names if more than one isolate. The presence of *qnr* genes, AR denoting antibiotic resistance with number of drugs resistant to, adhesion, LDH, and source among *Citrobacter* isolates were shown on the right. Note that for any STs with two isolates, properties for both were listed and separated by a comma. The tree was constructed using neighbor joining algorithm. ST and LDH indicate sequence types and lactate dehydrogenase, respectively. Cluster divisions are marked. Numbers on or near the nodes are bootstrap values from 1,000 replicates. Adhesion index: ***, >50; **, >1 and <50; *, <1; +/‐, ambivalent or no adhesion; ‐, no adhesion. Under LDH for cytotoxicity: H denotes highly cytotoxic if LDH values of >24%; M for intermediately cytotoxic if values from 18% to <24%; L for lowly or non-cytotoxic if LDH values from 1.4% to 17.8%. See **Table 2** for actual values.

The 46 *Citrobacter* isolates including 26 C*. freundii*, 6 C*. braakii*, and 14 C*. koseri* isolates were divided into 38 STs, with the 26 C*. freundii* isolates dividing into 22 STs, the 6 C*. braakii* isolates into 4 STs, and the 14 C*. koseri* isolates into 12 STs **(** **Table 2** and **Figure 2** **).** All of the 38 STs were novel STs in comparison to the STs in the public MLST database. Eight STs each contained two isolates with four *C. freundii* STs (ST434, ST441, ST451, and ST458), two *C. braakii* STs (ST435 and ST438), and two *C. koseri* STs (ST440 and ST455).

**Table 2:** Adherence, cytotoxicity, source, antibiotic resistance, genotypes, and antibiotic resistance phenotype of *Citrobacter* isolates.

Clusters and species	Isolates	Year	Source	STs	Adhesion	LDH	AR	Antibiotic resistance phenotype	*qnr*
Lineage I	C26	2017	Urine	443	+/-	21.9 ± 1.7	3	(AMP)(FOX)(AZM)	
*C. freundii*	C51	2018	Urine	460	+/-	19.4 ± 1.1	3	(AMP)(FOX)(AZM)	
	C43	2018	Sputum	456	**	18.7 ± 2.2	2	(FOX)(AZM)	
	C69	2018	Urine	471	*	10.7 ± 2.1	4	(AMP)(NA)(TE,DO)(SXT)	
	C37	2017	Urine	452	+/-	7.0 ± 1.6	6	(AMP)(FOX)(NA,CIP,LEV)(S,K)(SXT)(AZM)	
	C48	2018	Urine	458	–	5.8 ± 0.6	2	(FOX)(AZM)	
	C63	2018	Urine	458	–	21.1 ± 2.3	1	(FOX)	
	C36	2018	Sputum	451	+/-	20.4 ± 2.7	2	(AMP)(FOX)	
	C40	2018	Sputum	451	**	17.8 ± 1.0	3	(AMP)(FOX)(AZM)	
	C38	2018	Sputum	453	+/-	9.4 ± 0.7	4	(AMP)(CTX,FOX)(ATM)(CN)	
	C67	2018	Urine	469	–	4.8 ± 1.6	1	(FOX)	
	C53	2018	Urine	462	***	35.4 ± 2.4	2	(FOX)(AZM)	
	C23	2017	Urine	441	*	8.5 ± 1.8	2	(AMP)(AZM)	
	C57	2018	Sputum	464	*	18.0 ± 2.1	8	(AMP)(FOX)(NA)(S)(TE)(SXT)(AZM)	*qnrB 9*
	C24	2017	Urine	441	**	2.5 ± 0.8	7	(AMP)(CTX,FEP,FOX)(ATM)(NA)(CN,S)(SXT)(AZM)	*qnrB76*
	C77	2018	Urine	476	*	2.3 ± 0.1	5	(AMP)(CTX)(NA)(CN)(AZM)	*qnrB76*
	C27	2017	Urine	444	**	11.7 ± 1.1	5	(AMP)(CTX,CAZ,FOX)(IMP)(S)(AZM)	
	C35	2018	Sputum	450	+/-	22.1 ± 2.6	2	(AMP)(CTX,CAZ,FOX)	
	C72	2018	Urine	473	+/-	2.6 ± 0.6	4	(AMP)(FOX)(TE,DO)(SXT)	*qnrB11*
	C2	2014	Sputum	434	+/-	6.0 ± 0.5	9	(AMP)(CTX,CAZ,FOX)(ATM)(IMP)(NA,CIP,LEV)(CN,S,K)(TE,DO)(SXT)(AZM)	
	C9	2014	Urine	434	+/-	6.5 ± 1.1	8	(AMP)(CTX,CAZ,FOX)(MEM)(NA,CIP,LEV)(K)(TE,DO)(SXT)(AZM)	
	C28	2017	bile	445	–	12.8 ± 0.5	6	(AMP)(CTX,CAZ,FOX)(ATM)(IMP)(S,K)(AZM)	
	C58	2018	Urine	465	**	13.0 ± 1.4	3	(AMP)(FOX)(AZM)	
	C54	2018	Sputum	463	*	6.2 ± 1.6	1	(FOX/CFX)	
	C59	2018	bile	466	**	19.3 ± 2.0	3	(AMP)(CTX,CAZ,FOX)(ATM)	
	C62	2018	Urine	468	–	4.5 ± 0.5	6	(AMP)(FOX)(NA)(S)(SXT)(AZM)	
Lineage II	C52	2018	Sputum	461	**	12.1 ± 0.1	3	(AMP)(CTX,CAZ,FOX)(AZM)	
*C. braakii*	C19	2015	Sputum	439	**	6.2 ± 0.4	2	(AMP)(AZM)	
	C11	2014	Urine	435	**	8.4 ± 0.7	3	(AMP)(CTX,CAZ,FOX)(ATM)	
	C5	2014	Sputum	435	+/-	9.3 ± 1.1	5	(AMP)(CTX,CAZ,FOX)(ATM)(S)(AZM)	
	C17	2015	Secretion	438	**	12.9 ± 5.4	3	(FOX)(S)(AZM)	
	C18	2015	Secretion	438	**	6.1 ± 0.8	3	(AMP)(FOX)(AZM)	
Lineage III	C75	2018	Urine	475	*	1.4 ± 0.1	6	(AMP)(CTX,FEP)(CN,S)(C)(SXT)(AZM)	*qnrS1*
*C. koseri*	C81	2018	Sputum	478	**	7.1 ± 1.4	2	(AMP)(AZM)	
	C25	2017	Urine	442	***	9.1 ± 1.7	2	(AMP)(AZM)	
	C31	2017	Urine	447	***	14.1 ± 1.6	2	(AMP)(AZM)	
	C22	2017	Urine	440	*	10.9 ± 1.4	4	(AMP)(CTX,CAZ,FEP)(ATM)(AZM)	
	C73	2018	Urine	440	+/-	8.7 ± 1.7	1	(AMP)	
	C74	2018	Sputum	474	*	5.7 ± 1.2	3	(AMP)(S)(AZM)	
	C60	2018	Urine	467	–	10.9 ± 1.6	2	(AMP)(AZM)	
	C29	2017	Urine	446	***	13.0 ± 2.1	2	(AMP)(TE,DO)	*qnrS1*
	C39	2018	blood	454	***	20.6 ± 0.7	1	(AMP)	
	C33	2017	Sputum	448	**	12.8 ± 1.4	1	(AMP)	
	C41	2018	Sputum	455	**	17.7 ± 1.5	1	(AMP)	
	C42	2018	Urine	455	**	9.4 ± 0.7	2	(AMP)(AZM)	
	C44	2018	Urine	457	**	14.8 ± 0.8	1	(AMP)	

***, **, * correspond to adhesion index of >50, >1, and <50 and <1, respectively. +/- means ambivalent or no adhesion, - means no adhesion.; LDH (% ± SD): the lactate dehydrogenase released from HEp-2 cells; highly cytotoxic (>24%), intermediately cytotoxic (from 18% to <24%), lowly or non-cytotoxic (from 1.4% to 17.8%); ST, sequence types; AR, antibiotic resistance (number of drugs resistance); AMP, ampicillin; CTX, cefotaxime; CAZ, ceftazidime; FEP, cefepime; FOX, cefoxitin; ATM, aztreonam; IMP, imipenem; MEM, meropenem; NA, nalidixicacid; CLP, ciprofloxacin; LEV, levofloxacin; CN, gentamicin; AK, amikacin; S, streptomycin; K, kanamycin; TE, tetracycline; DO, doxycycline; SXT, trimethoprim/sulfamethoxazole; AZM, azithromycin.

The concatenated sequences of the seven housekeeping genes were used to construct a phylogenetic tree by the neighbor-joining algorithm to infer the relationship of the 46 *Citrobacter* isolates **(** **Figure 2** **)**. *Salmonella* LT2 was used as the outgroup. The tree could be divided into three lineages corresponding to species divisions with high bootstrap support. Lineage I, II, and III contained *C. freundii* isolates, *C. braakii* isolates, and *C. koseri* isolates, respectively. *Citrobacter* isolates from urine, sputum, and secretion samples were distributed among the different lineages **(** **Table 2** and **Figure 2** **)**.

**Figure 3:**
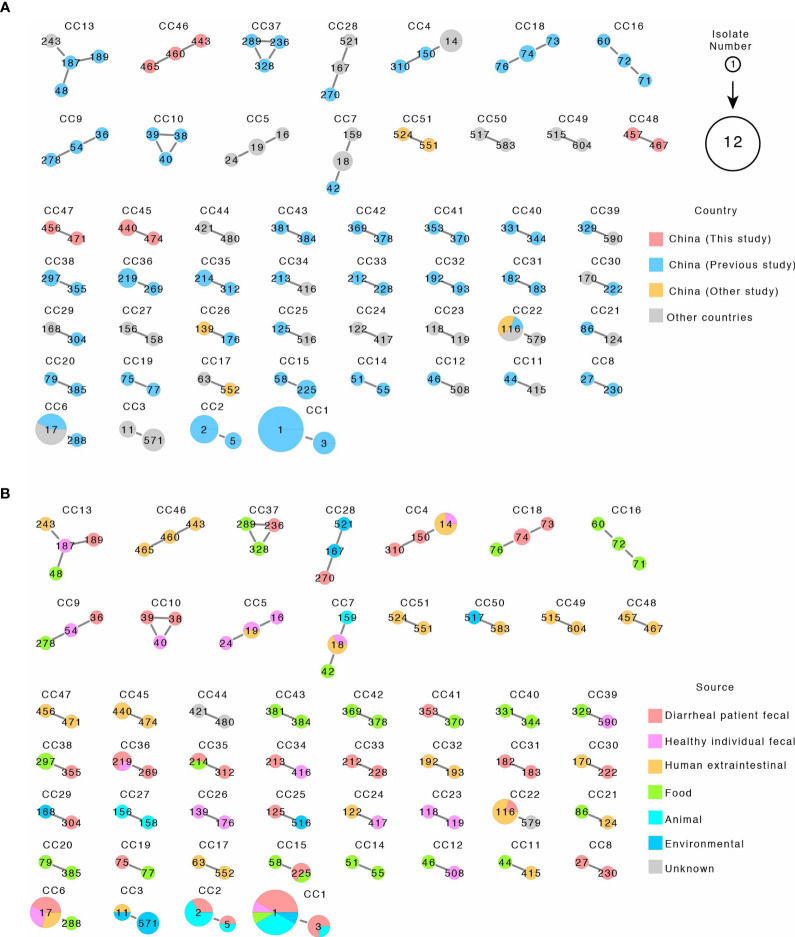
The 51 clonal complexes (CCs) by eBURST of 116 STs from this study and related STs from the public MLST database. **(A, B)** are the same CCs with A colored by country and B colored by source. Each circle represents an ST, and STs within CC are connected by solid line. ST numbers are marked inside the circle. Circle sizes are proportional to number of isolates. The pie chart within circles represented proportion of isolates from different country or sources as shown.

We further analyzed the 38 STs using the goeBURST algorithm to identify CCs. In this study, we defined CCs as clusters of STs differing by no more than one of the **s**even alleles to identify the most closely related STs. We computed CCs using our STs from this study and STs from the PubMLST database and identified 51 CCs including 4 CCs in this study and 47 CCs contained isolates from China and other countries (**Figure 3A** and **Supplementary Table S1**). The four CCs from this study (CC46–CC48) included nine STs from this study only and contained no isolates or STs from other Chinese studies or from other countries (**Figure 3** and **Supplementary Table S1**). All isolates of CC46 and CC48 were from urine samples, while CC45 and CC47 contained two isolates with one from urine and one from sputum, respectively (**Supplementary Figure S1**).

As shown in **Figure 3A** and **Supplementary Table S1**, some CCs were globally distributed and some CCs contained isolates from mixed sources including human clinical samples. CC6 contained two STs (ST288 and ST17). ST288 has one isolate from food, while ST17 has seven isolates with three isolated from diarrheal patients from China, two from healthy individual fecal samples in Latvia, and two from human extraintestinal samples in Poland. CC7 contained three STs (ST42, ST18, and ST159) and five isolates. The ST42 isolate was from food from China, the only ST159 isolate was from an animal source in Japan, while for the three ST18 isolates, two were from human extraintestinal samples in Poland and Spain, and one from healthy individual fecal samples in Greece. CC13 contained four STs each with one isolate which was found in China (three isolates) and USA, and the four isolates were separately from diarrheal patient fecal, healthy individual fecal, and human extraintestinal samples and food samples. CC4 contained three STs and six isolates, two isolates were from diarrheal patient fecal samples in China, and four isolates were from ST14 from other countries, including three human extraintestinal samples from Malaysia and one healthy individual fecal sample from Israel. CC22 contained two STs with five isolates and was found in China (three isolates) and other countries (two isolates from Thailand and one from The Netherlands) and with one from diarrheal patient fecal samples and four from human extraintestinal samples (blood, urine, and sputum) (**Figure 3** and **Supplementary Table S1**).

### Antibiotic Resistance of the *Citrobacter* Isolates and Prevalence of Multidrug Resistance

The 46 *Citrobacter* isolates were tested for susceptibility to 19 antibiotics belonging to 9 antibiotic classes using the disk diffusion method according to CLSI recommendations. The *C. freundii* isolates had higher antibiotic resistance rate than *C. braakii* and *C. koseri* isolates, although the number of isolates was small (**Table 3**). Most of the 26 C*. freundii* isolates were resistant to one or more of the β-lactam antibiotics, especially to penicillins (76.9%), cephalosporins (3.8–88.5%), monobactams (19.2%), and carbapenems (3.8–11.5%) (**Table 3**). For the four carbapenem resistant *C. freundii* isolates, three were resistant to IMP and one to MEM. Over half of the *Citrobacter* isolates (25/46) were MDR and were isolated from different years and sources (**Table 2** **)**. The *C. freundii* isolates from urine (11/16, 68.8%) showed higher rate of MDR than the *C. freundii* isolates from sputum samples with 4/8 (50%) being MDR **(** **Table 2** **)**.

**Table 3:** Prevalence of resistance to different antibiotics by species.

Antibiotic	*C. freundii* (n = 26)	*C. koseri* (n = 14)	*C. braakii* (n = 6)
	Resistance (%)	Resistance (%)	Resistance (%)
**Penicillins**			
Ampicillin	20 (76.9)	14 (100.0)	5 (83.3)
**Cephalosporins**			
Cefotaxime	9 (34.6)	2 (14.3)	3 (50.0)
Ceftazidime	6 (23.1)	1 (7.1)	3 (50.0)
Cefepime	1 (3.8)	2 (14.3)	0 (0)
Cefoxitin	23 (88.5)	0 (0)	5 (83.3)
**Monobactams**			
Aztreonam	5 (19.2)	2 (14.3)	2 (33.3)
**Carbapenems**			
Imipenem	3 (11.5)	0 (0)	0 (0)
Meropenem	1 (3.8)	0 (0)	0 (0)
**QUINOLONES**			
Nalidixicacid	8 (30.8)	0 (0)	0 (0)
Ciprofloxacin	3 (11.5)	0 (0)	0 (0)
Levofloxacin	3 (11.5)	0 (0)	0 (0)
**AMINOGLYCOSIDES**			
Gentamicin	4 (15.4)	1 (7.1)	0 (0)
Amikacin	0 (0)	0 (0)	0 (0)
Streptomycin	7 (26.9)	2 (14.3)	2 (33.3)
Kanamycin	4 (15.4)	0 (0)	0 (0)
**TETRACYCLINES**			
Tetracycline	5 (19.2)	1 (7.1)	0 (0)
Doxycycline	4 (15.4)	1 (7.1)	0 (0)
**SULFONAMIDES**			
SXT^#^	8 (30.8)	1 (7.1)	0 (0)
**MACROLIDES**			
Azithromycin	17 (65.4)	7 (50.0)	6 (100.0)
MDR^#^	17 (65.4)	3 (21.4)	5 (83.3)

^#^MDR (multidrug resistance): with resistance to at least one antibiotic of three or more distinct classes (MDR≥3). SXT, trimethoprim/sulfamethoxazole sulfafurazole.

### Prevalence of Fluoroquinolones Resistant Isolates

Among the 46 *Citrobacter* isolates, 8 C*. freundii* isolates were resistant to fluoroquinolones, all of which were resistant to NAL; 7 resistant to CIP and NOR; and 6 resistant to LEV (**Table 4**). Six fluoroquinolones resistant isolates were from urine and two from sputum, all of which were MDR. All of these eight NA-resistant isolates contained the mutation in codon 59 (Thr59Ile) in the *gyrA* gene. No mutation was found in the *parC* gene **(** **Table 4** **)**.

**Table 4:** Fluoroquinolone resistant values and alterations detected in the *gyrA* genes of *Citrobacter* isolates.

Isolates	Species	Year	Source	STs	MDR	NAL	CIP	LEV	NOR	*qnr*	*gyrA* mutation
C2	CF	2014	Sputum	434	9	>1024	16	8	32		Thr59Ile
C9	CF	2014	Urine	434	8	>1024	8	8	32		Thr59Ile
C24	CF	2017	Urine	441	8	512	8	4	16	*qnrB76*	Thr59Ile
C37	CF	2017	Urine	452	7	1024	256	64	128		Thr59Ile
C57	CF	2018	Sputum	464	8	512	4	4	8	*qnrB 9*	Thr59Ile
C62	CF	2018	Urine	468	7	128					Thr59Ile
C69	CF	2018	Urine	471	5	128	4		4		Thr59Ile
C77	CF	2018	Urine	476	5	1024	8	2	8	*qnrB76*	Thr59Ile

CF, C. freundii; NAL, nalidixicacid; CIP, ciprofloxacin; LEV, levofloxacin; NOR, norfloxacin.

### Prevalence of *qnrB* Genes

All the isolates were screened for *qnrA*, *qnrB*, *qnrS*, *qnrC*, *qnrD*, *aac(6’)-Ib-cr*, and *qepA* genes by PCR. One isolate was positive for *qnrS*, and four isolates were positive for *qnrB* (**Table 2**). PCR sequencing found that the *qnrB* alleles carried by the isolates were *qnrB9* (one isolate), *qnrB11* (one isolate), and *qnrB76* (two isolates). Two *qnrB76* harboring *C. freundii* isolates were all isolated from urine, all of which were resistant to NA, and were MDR. The *qnrB9* harboring *C. freundii* isolate was isolated from sputum which was resistant to NA and was MDR.

### Adherence and Cytotoxicity of *Citrobacter* Isolates

**Figure 4:**
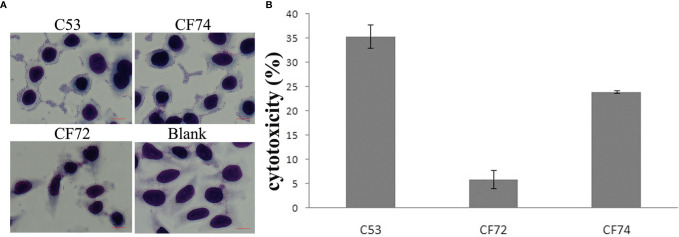
HEp-2 cell adhesion and cytotoxicity of *Citrobacter* isolates. **(A)** Light micrographs show the adherence patterns displayed by one strongly cytotoxic *Citrobacter* isolate (C53). Cells were stained with 1% Giemsa stain and examined under a light transmission microscope at a magnification of ×1,000. HEp-2 cell nuclei and bacteria were stained purple. *C*. *freundii* strain CF74 was used as highly adhesive control, *C. freundii* strain CF72 as lowly adhesive control, and Blank which had no bacteria as negative control. C53 displayed patterns of aggregative adherence to HEp-2 cells similar to CF74. Few bacteria were seen aggregated around HEp-2 cells in CF72. Bar: 10 µm. **(B)** Cytotoxicity of one highly cytotoxic *Citrobacter* isolate (C53) which was based on the LDH released from HEp-2 cells after exposure at 8 h. CF72 was used as lowly cytotoxic control strain and CF74 as highly cytotoxic strain. Y- axis is percentage of LDH released as measure of cytotoxicity.

We tested the *Citrobacter* isolates for adhesion and cytotoxicity to HEp-2 cells *in vitro* as done previously (Bai et al., 2012; Liu et al., 2017; Liu et al., 2018a; Liu et al., 2020). Five isolates showed high adhesion, with an adhesion index greater than 50, four of which belonged *C. koseri* and one *C. freundii.* Other isolates showed no to intermediate adhesion. Only one isolate released LDH more than 24% and was classified as highly cytotoxic, and nine isolates released LDH from 18% to <24% and were classified as intermediately cytotoxic, while the remaining 36 isolates showed LDH release from 1.4% to 17.8% and were lowly or non-cytotoxic (**Figures 4A, B ** and **Table 2**). When sample sources were considered, similar proportion of urine isolates (7/26, 26.7%) and sputum isolates (3/15, 20%) were highly/intermediately cytotoxic and/or highly adhesive **(** **Table 2****)**.

## Discussion

In this study, we surveyed *Citrobacter* extraintestinal infections in a hospital and found that *Citrobacter* isolates were mostly isolated from urine with 54.3% of the 46 isolates, followed by sputum samples (30.4%). Our findings were similar to those previously reported in India (Mohanty et al., 2007) and the USA (Hodges et al., 1978).

The 46 isolates were separated into 38 STs. The 38 STs from this study were compared with 566 STs from the *Citrobacer* MLST database, including isolates from all countries and sources. No ST in this study was shared with isolates from the database. In our previous study, 11 STs were shared with isolates from the database from other countries or regions or from different sources, and isolates of the same ST may be widely present in human fecal, food, and human extra intestinal samples (Liu et al., 2018a). We further analyzed the STs by CCs to ascertain any sharing of CCs and to determine whether there are any widely distributed CCs. The four CCs found in this study did not share any ST or CCs with other countries or other Chinese isolates. Fifteen CCs were found to be globally distributed. Some CCs contained human clinical isolates, including from diarrheal patient fecal samples and human extra-intestinal samples, which suggests that some CCs are more likely to cause diseases in humans.

A key strength of this study was that the population diversity and relationships of the isolates were assessed by MLST. The use of a standardized MLST scheme allowed comparison of data from this study with local and international MLST data from different sources. The combined MLST data from isolates from this study, isolates from human fecal samples and food samples from our previous studies, and other international isolates have revealed that there was no prevalent strains or clones, unlike many other bacterial pathogens such as UTI causing MDR *E. coli* ST131 with global distribution (Petty et al., 2014). However it is much needed of more studies using MLST or genome sequencing to better understand the genetic diversity and virulence of *Citrobacter* populations.

Clinical *Citrobacter* spp. strains are often resistant to multiple classes of antibiotics (Leski et al., 2016). Infections by MDR *Citrobacter* strains have been associated with a higher rate of in-hospital mortality compared to susceptible strains (Leski et al., 2016). Similarly, our study found that 54.3% of the isolates from extraintestinal infections were MDR with resistance to penicillin (84.8%), cephalosporins (67.4%), and azithromycin (65.2%), but susceptible to carbapenems (87.0%).

Carbapenem resistant Enterobacteriaceae (CRE) has become a major public health threat that requires urgent attention (Ramsamy et al., 2020). Carbapenem-resistant *Citrobacter* spp. isolates have been reported due to the acquisition of worldwide disseminated carbapenemases, such as New Delhi Metallo-β-lactamase (NDM), VIM-1, OXA-48, KPC-2, and VIM-2 (Hammerum et al., 2016; Arana et al., 2017; Faccone et al., 2019; Schweizer et al., 2019; Gobeille Paré et al., 2020; Räisänen et al., 2021). *bla*_NDM-1_-positive *C. freundii* has been increasingly reported in China, India, Denmark, and South Africa (Yang et al., 2018) and VIM-1- and VIM-2-positive *C. freundii* have also been reported in Europe (Gaibani et al., 2013; Porres-Osante et al., 2014; Santos et al., 2017). In this study, four isolates were resistant to IMP or MEM. We did not determine the molecular mechanisms of carbapenem resistance of these four isolates which will be done in future studies. In our previous study, all isolates which were isolated from food, diarrheal patient fecal, healthy individual fecal, and environmental samples were susceptible to carbapenems (Liu et al., 2017; Liu et al., 2018a; Liu et al., 2020).

The prevalence of quinolone resistance and mutations of quinolone resistance genes varied among *Citrobacter* isolates. In our previous study, *C. braakii* had the highest proportion of quinolone resistant isolates (52.6%), followed by *C. freundii* with 23.7% (Liu et al., 2020). In this study, *C. freundii* had the highest proportion of quinolone resistant isolates. *Citrobacter* isolates with mutations in the quinolone resistance determining region of *gyrA*, including Thr83Ile and Asp87Asn, have shown reduced susceptibility to fluoroquinolones (Weigel et al., 1998; Minarini and Darini, 2012). In our previous studies (Liu et al., 2018a; Liu et al., 2020), four quinolone resistant *C. freundii* isolates had mutations in Thr59Ile, Gln111Arg, and Ile134Val. Twenty-seven quinolone resistant isolates carried mutations in Thr59Ile and one having three mutations in Thr59Ile, Gln111Arg, and Ile134Val. In this study, among eight quinolone resistant *C. freundii* isolates, all had the Thr59Ile mutation in the *gyrA* gene.

Cytotoxicity and adhesive ability *in vitro* were assessed for all isolates, which varied widely. Among the 50 isolates, only five and one were shown to be highly adhesive and highly cytotoxic, respectively. We did not find any association of cytotoxicity and adhesive ability with the source of the isolates (urine or sputum samples). Since all isolates were from clinical infections, it seems that *in vitro* cytotoxicity and adhesive ability of an isolate may not be indicative of their disease causing ability. However, the numbers of isolates were small and there were no patient data to determine whether any of these parameters is suggestive of more severe disease outcomes.

## Conclusion

We analyzed 46 extraintestinal clinical *Citrobacter* isolates (26 C*. freundii*, 6 *C. braakii*, and 14 C*. koseri* isolates) from 2014 to 2018 in Maanshan people’s hospital of Anhui Province, China. The isolates showed high diversity with 38 STs, all of which were novel STs. Nine of the 38 STs belonged to four CCs, but no isolates or STs from this study shared the same CCs with isolates from other countries or other Chinese isolates reported. MDR was prevalent among the isolates causing extraintestinal infections at 54.3%, and four isolates (8.7%) were carbapenem resistant (IMP or MEM). All eight quinolone resistant *C. freundii* isolates carried the Thr59Ile mutation in the *gyrA* gene. Only a small proportion of the isolates were found to be highly cytotoxic and adhesive with no correlation to sample sources. This study has shed more light on the genetic diversity and antibiotic resistance of extraintestinal infection causing *Citrobacter* in China.

## Data Availability Statement

The original contributions presented in the study are included in the article/**Supplementary Material**. Further inquiries can be directed to the corresponding authors.

## Ethics Statement

The studies involving human participants were reviewed and approved by the Ethical Committee of the National Institute for Communicable Disease Control and Prevention, Chinese Center for Disease Control and Prevention, China (No. ICDC-2016007). The patients/participants provided their written informed consent to participate in this study.

## Author Contributions

LL and JX designed the project. YW and LZ carried out the sampling work. HZ, MY and HS carried out the experiments. LL, RL, and DH analyzed data. LL and RL drafted the manuscript. All authors contributed to the article and approved the submitted version.

## Funding

This work was supported by the National Key Research and Development Program of China (2019YFC1200505 and 2019YFC1200500) and grants from National Natural Science Foundation of China (No. 81301401).

## Conflict of Interest

The authors declare that the research was conducted in the absence of any commercial or financial relationships that could be construed as a potential conflict of interest.

## Publisher’s Note

All claims expressed in this article are solely those of the authors and do not necessarily represent those of their affiliated organizations, or those of the publisher, the editors and the reviewers. Any product that may be evaluated in this article, or claim that may be made by its manufacturer, is not guaranteed or endorsed by the publisher.
