# Supplementary figures and images for: Antimicrobial Resistance and Molecular Characterization of Citrobacter spp. Causing Extraintestinal Infections
Source: Front Cell Infect Microbiol. 2021 Aug 27;11:737636. doi: 10.3389/fcimb.2021.737636 (PMC8429604; doi:10.3389/fcimb.2021.737636)

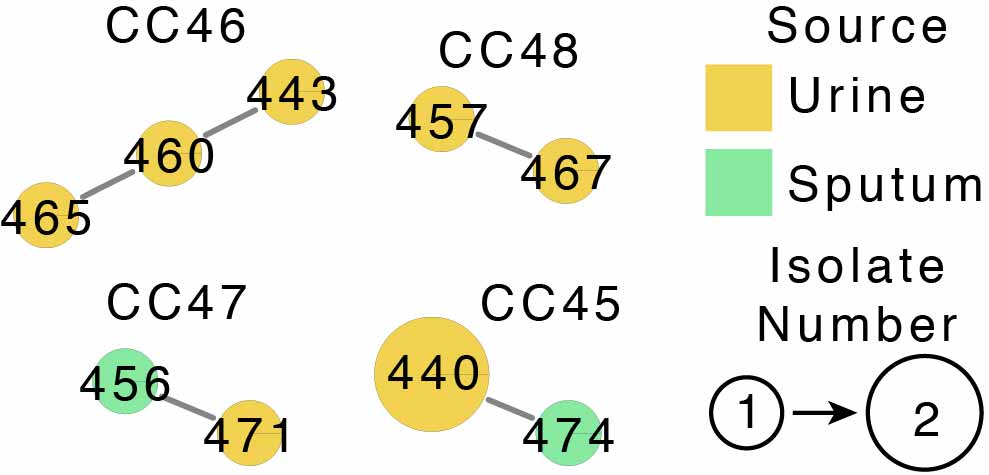

Supplement: Supplementary file 1 [file Image_1.jpeg]
