# Supplementary material for: Antimicrobial Resistance and Molecular Characterization of Citrobacter spp. Causing Extraintestinal Infections
Source: Front Cell Infect Microbiol. 2021 Aug 27;11:737636. doi: 10.3389/fcimb.2021.737636 (PMC8429604; doi:10.3389/fcimb.2021.737636)
Supplement: Supplementary file 3 [file Table_2.docx]

**Figure S1.** The four clonal complexes by eBURST of nine STs from this study. Isolates from different source are shown as different colors and STs are marked inside the circle. Circle sizes are proportional to number of isolates.
